# Resonant frequency of gold/polycarbonate hybrid nano resonators fabricated on plastics via nano-transfer printing

**DOI:** 10.1186/1556-276X-6-90

**Published:** 2011-01-17

**Authors:** Edward Dechaumphai, Zhao Zhang, Nathan P Siwak, Reza Ghodssi, Teng Li

**Affiliations:** 1 Department of Mechanical Engineering, University of Maryland, College Park, MD 20742, USA; 2 MEMS Sensors and Actuators Laboratory (MSAL), Department of Electrical and Computer Engineering, University of Maryland, College Park, MD 20742, USA; 3 Institute for Systems Research, University of Maryland, College Park, MD 20742, USA; 4 Maryland NanoCenter, University of Maryland, College Park, MD 20742, USA

## Abstract

We report the fabrication of gold/polycarbonate (Au/PC) hybrid nano resonators on plastic substrates through a nano-transfer printing (nTP) technique, and the parametric studies of the resonant frequency of the resulting hybrid nano resonators. nTP is a nanofabrication technique that involves an assembly process by which a printable layer can be transferred from a transfer substrate to a device substrate. In this article, we applied nTP to fabricate Au/PC hybrid nano resonators on a PC substrate. When an AC voltage is applied, the nano resonator can be mechanically excited when the AC frequency reaches the resonant frequency of the nano resonator. We then performed systematic parametric studies to identify the parameters that govern the resonant frequency of the nano resonators, using finite element method. The quantitative results for a wide range of materials and geometries offer vital guidance to design hybrid nano resonators with a tunable resonant frequency in a range of more than three orders of magnitude (e.g., 10 KHz-100 MHz). Such nano resonators could find their potential applications in nano electromechanical devices. Fabricating hybrid nano resonators via nTP further demonstrates nTP as a potential fabrication technique to enable a low-cost and scalable roll-to-roll printing process of nanodevices.

## Introduction

Flexible electronics is an emerging technology that will have a significant social impact through an exciting array of applications, such as low-cost electronic paper, printable thin-film solar cells, and wearable power harnessing devices, to name a few [1-7]. Future success of flexible electronics hinges upon new choices for fabrication processes that are cost-effective, scalable to large areas, and compatible with both organic and inorganic materials [8]. Roll-to-roll printing of flexible devices allows for dramatic reduction in capital and device costs, resulting in lightweight, thin, rugged, and large area flexible devices [9]. While this promising technology still being in its infancy, there are existing efforts to explore enabling printing technology for roll-to-roll process, such as ink-jet printing [10], micro-contact printing (μCP) [11,12], and nano-transfer printing (nTP) [13-19]. Unlike inkjet printing and μCP, nTP is inherently compatible with nano-scale features and the resulting devices are as good as those fabricated via traditional processing methods [17]. nTP primarily relies on differential adhesion for the transfer of a printable layer from the transfer substrate to a device substrate. Various organics and inorganics can be printed in the same manner thus avoiding mixed processing methods and allowing multilayer registration. So far, nTP has been successfully used to fabricate a range of functional components for flexible devices, such as organic thin-film transistors (OTFTs) [17], carbon nanotube TFTs [20], graphene TFTs [16,21], and inductors. In this article, we report the fabrication of gold/polycarbonate (Au/PC) hybrid mechanical nano resonators on plastic substrates through an nTP process, and the parametric study of the resonant frequency of the resulting hybrid nano resonators.

**Figure 1:**
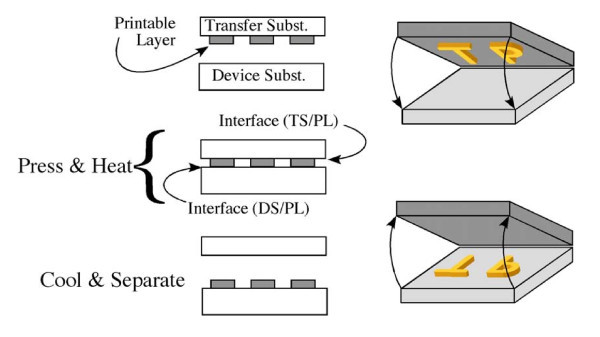
**Schematics of the nTP process**.

The nTP process has been described in detail elsewhere [15,17] and is briefly described here and illustrated in Figure 1. The first step was to prepare a printable layer on the surface of a transfer substrate. The second step was to sandwich the printable layer in between the transfer and device substrates. The third step was to apply pressure such that the printable layer was in contact with both substrates. As long as the adhesion of the printable layer to the device substrate is larger than to the transfer substrate, upon separation of the substrates, the printable layer will remain in contact with the device substrate and thus have been successfully transfer printed. If the transfer substrate is a thermoplastic or has a surface containing a thermally activated adhesion layer, then the application of temperature can be used to increase the needed differential adhesion. nTP has been applied as a means of fabricating thin-film transistors on plastic substrates. Previous study has demonstrated high quality transistor devices incorporating small molecule organic (penatcene), polymeric organic (P3HT), inorganic (Si ribbons), and carbon-based (both carbon nanotubes and graphene) semiconductor materials [15,17,20-25]. These devices also have incorporated previously printed Au source/drain and gate electrodes separated by a (printed) polymer dielectric layer.

**Figure 2:**
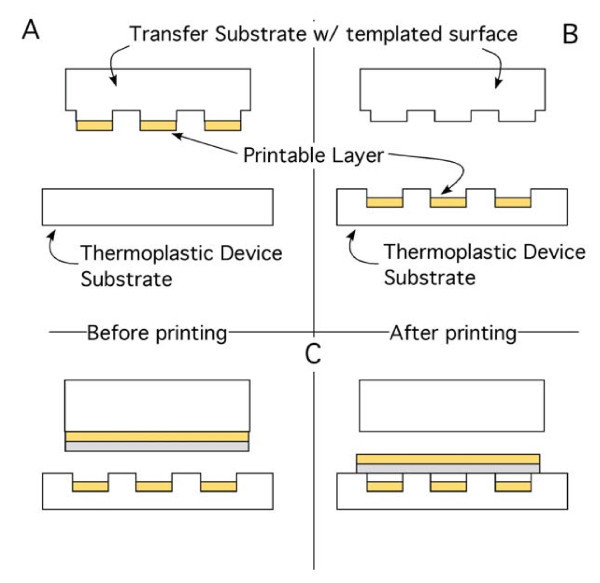
**Illustration of the fabrication of a three-dimensional device via nTP**. A templated surface containing a printable layer on the raised portion of a transfer is shown in **(a) **before and **(b) **after printing onto a thermoplastic device substrate. The sequential printing of a multilayer printable layer is shown in **(c)**.

**Figure 3:**
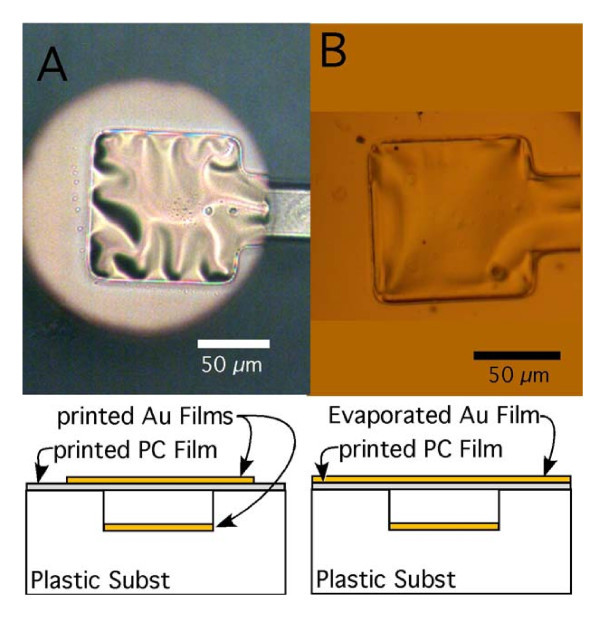
**Optical images of Au/PC hybrid nano resonators printed onto a PC substrate with the top Au film**. **(a) **printed along with the top PC film and **(b) **vacuum deposited after printing of the top PC film.

If the transfer substrate contains a templated surface in addition to a printable layer as illustrated in Figure 2a, then the nTP process can be used to create three-dimensional structures on the device substrate, which contain the printed materials as is illustrated in Figure 2b. The fabrication of such nanostructures as mechanical resonators, microfluidic, and MEMS/NEMS devices can be accomplished by assembling sequentially printed materials on the device substrate as illustrated in Figure 2c. As a demonstration of the concept, the mechanical resonators shown in Figure 3 have been fabricated by printing Au and PC membranes over previously printed/templated Au electrodes embedded within cavities on a PC substrate. The detailed fabrication of these mechanical resonators is presented as follows.

A 200-500-nm-thick Au printable layer was fabricated on a Si transfer substrate using standard photolithography, followed by metals deposition using an e-beam deposition system and lift-off. The resulting Au pattern was used as an etch mask such that the Si transfer substrate was etched to a depth of approximately 8 μm in an RIE chamber using 20 SCCM SF_6_, 20 mTorr, and 100 W. The Au printable layer covering the raised portion of the templated transfer substrate was printed onto a PC device substrate in a Nanonex NX2500 nano-imprintor at 160°C and 500 psi for 3 min. A second transfer substrate was prepared by performing metals deposition of a 35-nm Au film through a shadow mask onto a Si transfer substrate and then spin coating a 200-nm thick PC film over the Au film. The Au/PC membrane was transfer printing over the previously printed PC substrate at 130°C and 500 psi for 3 min. Note that the first printing temperature is above the glass transition temperature (*T*_g_) of the PC substrate while the second printing temperature is below *T*_g_. The higher temperature was used to ensure that the templated surface was fully replicated into the surface of the PC substrate while the lower temperature was used to ensure that the templated surface of the PC substrate was retained. The resulting mechanical resonator is shown in Figure 3a. Note that this device exhibits wrinkles in the top layer Au/PC membrane. Such features result from the compressive strain built up within the Au membrane due to the differential thermal expansion between the Au and PC materials. Figure 3b shows a similar device where the Au membrane was deposited near room temperature in an e-beam evaporator rather than transfer printed at 130°C. Note that the device containing the directly deposited Au film has notable fewer wrinkles than the device containing the printed Au film.

A preliminary measurement of the resonant frequency on these devices was performed visually under an optical microscope. The top and bottom electrodes were contacted using probe tips connected to a square wave AC voltage source. A voltage of approximately 100 V was applied across the electrodes as a means to mechanically excite the devices and the frequency swept from 400 to 600 KHz for the device in Figure 3a and from 10 to 35 KHz for the device in Figure 3b. The optical microscope was initially in focus on the surface of the Au/PC film. As the frequency of the applied voltage reaches the resonant frequency of the nano resonator, the Au/PC film is exited and starts to vibrate. As a result, the surface of the Au/PC film in the microscope becomes out of focus. The frequency as a change in focus of the Au/PC film surface was recorded as the resonate frequency. In this way, the resonant frequencies were estimated to be 520 and 25 KHz, respectively. It is expected that the resonant frequency of a hybrid nano resonator depends on both the geometric parameters of the design (i.e., the width of the cavity over which Au/PC is fabricated, the thickness of the Au/PC film) and the mechanical properties of the constituent materials (i.e., elastic moduli of Au and PC). For example, a similar nano resonator fabricated over a narrower cavity has a higher resonant frequency, with all other parameters remaining the same.

To guide further experiments and explore the design limit of hybrid nano resonators fabricated via nTP, we next perform systematic parametric studies to investigate the effects of aforementioned governing parameters on the resonant frequencies of hybrid nano resonators, using finite element analysis. Specifically, we study the effects of the PC thickness, the cavity width, and the elastic modulus of the polymeric film (e.g., if a polymer different from PC is used). The results from the parametric studies can serve as guidelines to design hybrid nano resonators with tunable resonant frequencies.

**Figure 4:**
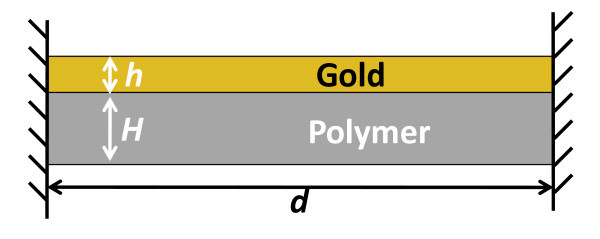
**Schematics of the computational model of the hybrid nano resonator**. Here, *h *= 35 nm; *H *and *d *are varied in parametric study.

Given that the plastic substrate is significantly thicker than the Au/PC bilayer (e.g., more than thousands times) and the bottom Au film is well adhered to the bottom of the cavity in the plastic substrate, the resonant vibration of the hybrid nano resonator shown in Figure 3 can be reasonably assumed to occur mainly in the freestanding portion of the Au/PC bilayer. Above said, we simplify the model of the hybrid nano resonator as a bilayer structure consisting of a thin Au film of thickness *h *that is well bonded to a polymeric film of thickness *H*, as illustrated in Figure 4. The two ends of the bilayer are clamped, which is justified given the large ratio of the cavity width over the bilayer thickness. Here we assume the Au/PC bilayer is fabricated over an infinitely long cavity of width *d*; therefore, the resonant vibration of the Au/PC bilayer can be assumed to be in plain strain condition. The effect of such an assumption will be further discussed later in the article.

The finite element code, ABAQUS 6.9, was used to compute the natural frequencies of the resonator models. In the finite element model, the top surface of the polymeric film was tied with the bottom surface of the Au film. Therefore, no delamination between the Au and the polymeric film occurs. Both Au and polymer are modeled as homogenous and elastic solids. The material properties of Au and PC used in the model are listed in Table 1. Four-node bilinear elements with reduced integration are used for both the Au and the polymer film. Particular efforts were placed on meshing to guarantee sufficient mesh density and suitable element aspect ratio to achieve satisfactory computation precision.

**Table 1:** Material properties used in computational model

	Gold	PC
Elastic modulus (GPa)	78	2
Poisson's ratio	0.44	0.37
Density (kg/m^3^)	19.3 × 10^3^	1.2 × 10^3^

In the parametric studies, we fixed the thickness and the elastic modulus of the Au film to be 35 nm and 78 GPa, respectively. The thickness of the polymeric film *H *was varied between 0.2 and 10 μm and the elastic modulus of the polymeric film *E *was varied between 10 MPa and 10 GPa (e.g., corresponding to a range from a compliant elastomer film to a stiff plastic film). The cavity width *d *is varied between 5 and 50 μm. The resonant frequency analysis was carried out via eigenmode and eigenvalue extraction using Lanzcos method in ABAQUS 6.9.

**Figure 5:**
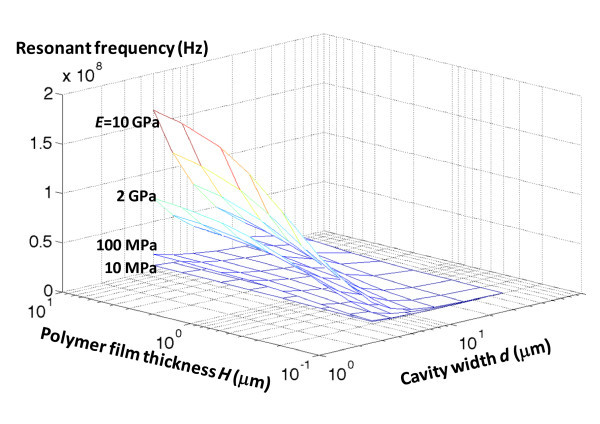
**Resonant frequency of the base mode of a hybrid nano resonator as a function of the thickness of the polymer film *H *and the cavity width *d*, for various stiffnesses of the polymer film *E***. Note the logarithmic scales for both *H *and *d*.

Figure 5 plots the resonant frequencies of the base eigenmode of the hybrid nano resonators in the parameter space spanned by the cavity width and the thickness of the polymer film, for various elastic moduli of the polymer film. For a given elastic modulus of the polymer film, the resonant frequency increases monotonically as the substrate thickness increases and the cavity width decreases. Such an increase in resonant frequency becomes rather prominent when a stiff plastic film is used in the nano resonator (e.g., high elastic modulus). For example, for *E *= 10 GPa, the resonant frequency can be as high as 91 MHz when *H *= 1 μm and *d *= 5 μm. By contrast, for *E *= 10 MPa, the resonant frequency can be as low as 23.2 KHz when *H *= 0.2 μm and *d *= 50 μm. In other words, there is significant tunability (e.g., more than three orders of magnitude) of the resonant frequency of the base mode of the hybrid nano resonators within the parameter space we explored.

**Figure 6:**
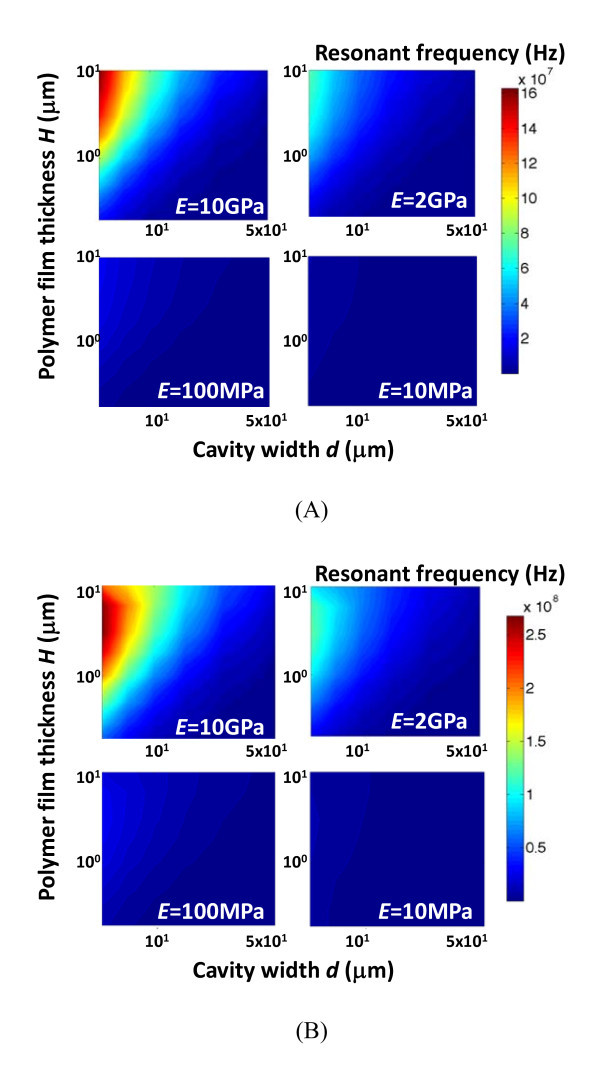
**Contour plots of the resonant frequencies of (a)**. the base and **(b) **secondary modes of the hybrid nano resonators as a function of the thickness of the polymer film *H *and the cavity width *d*, for various stiffnesses of the polymer film *E*.

Figure 6 compares the contour plots of the resonant frequencies of the base and secondary modes of the hybrid nano resonators as a function of the cavity width and the thickness of the polymer film, for various elastic moduli of the polymer film. For a given combination of *d*, *H*, and *E*, the resonant frequency of the secondary mode is higher than that of the base mode. For example, the secondary mode resonant frequency is 199 MHz when *H *= 1 μm, *d *= 5 μm, and *E *= 10 GPa, compared with the base mode resonant frequency of 91 MHz. As shown in Figure 6b, the secondary mode resonant frequency increases monotonically as *H *increases when the cavity width is relatively large (e.g., *d *>10 μm), but reaches its maximum at a certain value of *H *then decreases as *H *increases when *d *is small. Similar trends were also observed in the simulation results of higher order resonant modes. Such a dependence of higher order mode resonant frequency on *H *and *d *can be explained as follows. When *H *and *d *become comparable (e.g., a thick polymer film over a narrow cavity), the resulting nano resonator does not depict a thin-film profile. As a result, the higher order eigenmodes of such a nano resonator assume irregular modal shapes that are different from the regular sinusoidal modal shapes of a thin-film nano resonator. Other parameters, such as the boundary conditions at the two ends, come into play in determining the resonant frequency. Nonetheless, the dependence of the base mode resonant frequency on *H *and *d*, which is of the most technical significance in practice, is monotonic within the parameter space we explored.

In our parametric studies, our simulation models correspond to a hybrid nano resonator fabricated over an infinitely long cavity. Compared to that fabricated over a square cavity (e.g., Figure 3), our simulation model ignores the mechanical constraint imposed by another two sides of the cavity to the Au/PC bilayer. In this sense, our simulation results underestimate the resonant frequencies of nano resonators fabricated in our experiments. For example, the predicted base mode resonant frequency is 119 KHz for *H *= 0.2 μm and *d *= 50 μm, which falls in between the two measured resonant frequencies (520 and 25 KHz, respectively). Further measurement of the resonant frequencies of the hybrid nano resonators at higher precision is under exploration and will be reported elsewhere. In our simulations, the wrinkles in the Au films due to thermal mismatch during nTP process are not considered. Wrinkles in the Au film lead to increased bending resistance of the nano resonator, therefore result in a resonant frequency higher than that of a smooth nano resonator. In this sense, the simulation results underestimate the resonant frequency of the Au/PC nano resonators. Recent study shows that the interfacial defects also affect the quality of nTP process [19]. For example, an interfacial delamination along the interface between transfer substrate and printable layer (Figure 1) is beneficial, while that along the interface between printable layer and device substrate is detrimental for the success of nTP process. Such understandings can be indeed leveraged to enhance the quality of nTP processes, such as by introducing pre-delamination along the desirable interface via controlled adhesion. We will explore such a strategy in future works to further improve the yield of the nano resonator fabrication.

In summary, we fabricated Au/PC hybrid mechanical nano resonators on plastic substrates through an nTP process, and conducted systematic computational studies to decipher the geometric parameters and mechanical properties that govern the resonant frequency of the resulting hybrid nano resonators. We showed that the hybrid nano resonators can be mechanically excited when the frequency of the applied AC voltage reaches the resonant frequency of the hybrid nano resonators. The quantitative results for a wide range of materials (from PC to elastomers) and geometries offer vital guidance to design hybrid nano resonators with a tunable resonant frequency in a range of more than three orders of magnitude (e.g., 10 KHz-100 MHz). Given the versatility of nTP process, it is reasonable to expect that such designs of nano-scale resonators can be achieved. While the exploration reported in this article is still preliminary, there is no doubt that such hybrid nano resonators could find their potential applications in nano- electromechanical devices. Fabricating hybrid nano resonators via nTP further demonstrates nTP as a potential fabrication technique to enable a low-cost and scalable roll-to-roll printing process of nanodevices.

## Abbreviations

Au/PC: gold/polycarbonate; μCP: micro-contact printing; nTP: nano-transfer printing; OTFTs: organic thin-film transistors.

## Competing interests

The authors declare that they have no competing interests.

## Authors' contributions

TL and RG designed research; ED, ZZ, and TL conducted modeling research; NPS and RG performed experimental research; TL, EG, ED, ZZ, and NPS analyzed data; and TL, ED and ZZ wrote the paper.
